# Supplementary material for: Detailed molecular and epigenetic characterization of the pig IPEC-J2 and chicken SL-29 cell lines
Source: iScience. 2023 Feb 20;26(3):106252. doi: 10.1016/j.isci.2023.106252 (PMC10018572; doi:10.1016/j.isci.2023.106252)
Supplement: Data S2. Complete homer output for identified motifs in Chicken SL-29, related to Tables 5 and 6 — Homer motif analysis results for histone modifications H3K4me1, H3K4me3, H3K27ac, enhancers, and ATAC-seq of chicken SL-29 cell line. Parameters for possible false positives is as mentioned earlier for S5. [file mmc3.zip › Data_S2/S6/Chicken_SL_29/peak_files_H3K27ac/homerResults/motif14.similar.html]

motif14

## Information for motif14

A
T
G
C
A
C
G
T
A
T
C
G
C
A
G
T
A
C
T
G
C
G
T
A
C
T
G
A
C
T
G
A
  
Reverse Opposite:  

G
A
C
T
G
A
C
T
G
C
A
T
T
G
A
C
G
T
C
A
T
A
G
C
T
G
C
A
T
A
C
G
  

|  |  |
| --- | --- |
| p-value: | 1e-31 |
| log p-value: | -7.281e+01 |
| Information Content per bp: | 1.499 |
| Number of Target Sequences with motif | 10595.0 |
| Percentage of Target Sequences with motif | 35.13% |
| Number of Background Sequences with motif | 9653.9 |
| Percentage of Background Sequences with motif | 31.94% |
| Average Position of motif in Targets | 149.6 +/- 84.7bp |
| Average Position of motif in Background | 149.8 +/- 88.3bp |
| Strand Bias (log2 ratio + to - strand density) | -0.0 |
| Multiplicity (# of sites on avg that occur together) | 1.25 |
| Motif File: | file (matrix) reverse opposite |

### Similar de novo motifs found

|  |  |  |  |  |  |  |  |
| --- | --- | --- | --- | --- | --- | --- | --- |
| Rank | Match Score | Redundant Motif | P-value | log P-value | % of Targets | % of Background | Motif file |
| 1 | 0.605 | T G C A T A G C C T A G A C T G C T A G C T A G C T G A C G T A T C G A T G C A | 1e-27 | -63.657771 | 25.88% | 23.18% | motif file (matrix) |
